# CC16-TNF-α negative feedback loop formed between Clara cells and normal airway epithelial cells protects against diesel exhaust particles exposure-induced inflammation

**DOI:** 10.18632/aging.203356

**Published:** 2021-08-02

**Authors:** Ting Hu, Fenglan Sun, Xinjuan Yu, Qinghai Li, Long Zhao, Wanming Hao, Wei Han

**Affiliations:** 1 Qingdao Municipal Hospital, School of Medicine, Qingdao University, Qingdao 266011, China

**Keywords:** diesel exhaust particles, clara cell secretory protein, C/EBPα, Munc18b, TNF-α

## Abstract

CC16 is almost exclusively expressed in non-ciliated epithelial Clara cells, and widely used as a Clara cell marker. Diesel exhaust particles (DEPs), the fine particulate matters produced by diesel engines, cause or exacerbate airway-related diseases. Our previous study documented that DEP inhibits the CC16 expression in the immortalized mouse Clara cell line through methylation of C/EBPα promoter. However, the molecular mechanism by which DEP regulates CC16 secretion is unclear. Here, we isolated CC16 containing Clara cells (CC16^+^) from human distal lung, and found that DEP inhibited CC16 secretion from CC16^+^ cells via methylation of C/EBPα and inhibition of Munc18b transcription. CC16^+^ cell conditioned media containing different concentrations of CC16 was prepared and used for culture of airway epithelial cells BEAS-2B with no expression of CC16. A positive correlation was observed between CC16 level and DEP-induced autophagy activity, and a negative correlation between CC16 level and DEP-induced pro-inflammatory cytokine TNF-α, IL-6, and IL-8 level, suggesting that CC16 might mitigate DEP-induced inflammation via promoting autophagy in BEAS-2B cells. This result was further confirmed by adding recombinant CC16 to BEAS-2B cells exposed to DEP. Moreover, CC16 level was significantly increased when CC16^+^ cells were cultured in BEAS-2B cell conditioned medium containing TNF-α or the normal medium supplemented with recombinant TNF-α, suggesting that TNF-α induced CC16 production and secretion from CC16^+^ cells. Collectively, these data point that CC16 and TNF-α form a negative feedback loop, and this negative feedback loop between Clara cells and normal airway epithelial cells protects against DEP exposure-induced inflammation.

## INTRODUCTION

Accumulating epidemiological, laboratory and clinical studies demonstrate that exposure to increased level of particulate matter (PM) is related to the increased morbidity and mortality of multiple human diseases, such as allergic disorders, respiratory diseases, cardiac diseases, and renal diseases [1–4]. In recent years, PM2.5, less than 2.5 μm in diameter, has received significant attention. Due to the small size, PM2.5 is able to reach alveolar spaces, and precipitates throughout the whole lung, including the periphery, thereby triggering the biological responses and eliciting diseases [5, 6].

With the development of the economy, logistics based on the transportation industry has become a major service industry in the modern national economy. In the transport industry, diesel engines are predominantly used due to high efficiency and low cost. Diesel exhaust particles (DEPs), the fine particulate matters produced by diesel engines, are one of the major parts of PM2.5. There is growing evidence that DEPs induce or exacerbate the diseases involving the airway, such as pulmonary arterial hypertension, asthma, and chronic obstructive pulmonary disease (COPD) [7–9].

Our previous *in vivo* and *in vitro* experiments also proposed that DEP exposure may impair lung function through inhibiting of CC16 expression by methylation of the C/EBPα promoter [10]. CC16, a secretory protein, functions after secretion from cells. However, the molecular mechanism by which DEP regulates the secretion of CC16 is uncertain. Munc18b is a limiting component of the exocytic machinery of the secretory epithelia and immune cells. Our previous study displayed that Munc18b knockdown significantly inhibited the MUC5AC secretion from bronchial epithelial cells [11, 12]. C/EBPα, a transcription factor, regulates the expression of multiple genes by binding to the promoter. By searching the Jaspar database, we found that the potential binding motifs exist between C/EBPα and the Munc18b promoter region in different species (human, rat and mouse). Given these findings, we further explored whether C/EBPα promotes CC16 secretion via regulation of Munc18b expression.

CC16, specifically expressed in distal airway Clara cells, is the most abundant secretory protein in the airway surface fluid, accounting for 5% of the total protein quantity recovered by bronchoalveolar lavage [13]. It is well known that CC16 has anti-inflammatory and antioxidant effects [14–19]. The change in CC16 level not only has a profound effect on the composition of the airway surface fluid, but also affects the response of the airway epithelium to environmental stimuli [20–22]. For instance, CC16 mitigates house dust mite-induced airway inflammation and damage via regulation of airway epithelial cell apoptosis [20]; and CC16 level is also correlated with cigarette smoke exposure in bronchial epithelial cells, and protects against lung injury in smokers [21]. It has been widely reported that DEP causes a pro-inflammatory response in bronchial epithelial cells [23–25]. However, the role of CC16 in bronchial epithelial cells exposed to DEP has not been reported. We speculated that CC16 protects against bronchial epithelial cell inflammation, and the lower CC16 secretion level from Clara cells caused by DEP exposure exerts the weaker protective effects in DEP-induced bronchial epithelial cell inflammation.

Additionally, TNF-α has been reported to drive CC16 production [26]. Given these findings, it was tempting to speculate that DEP-induced TNF-α increase in bronchial epithelial cells stimulates CC16 production by Clara cells, and CC16 and TNF-α form a negative feedback, thereby inhibiting DEP exposure-induced injury.

Herein, we aimed to investigate whether DEP inhibits CC16 secretion from Clara cells via C/EBPα-Munc18b axis, and CC16 and TNF-α form a negative feedback against DEP-induced bronchial epithelial cell inflammation.

## RESULTS

### Characterization of the sorted cells

**Figure 1:**
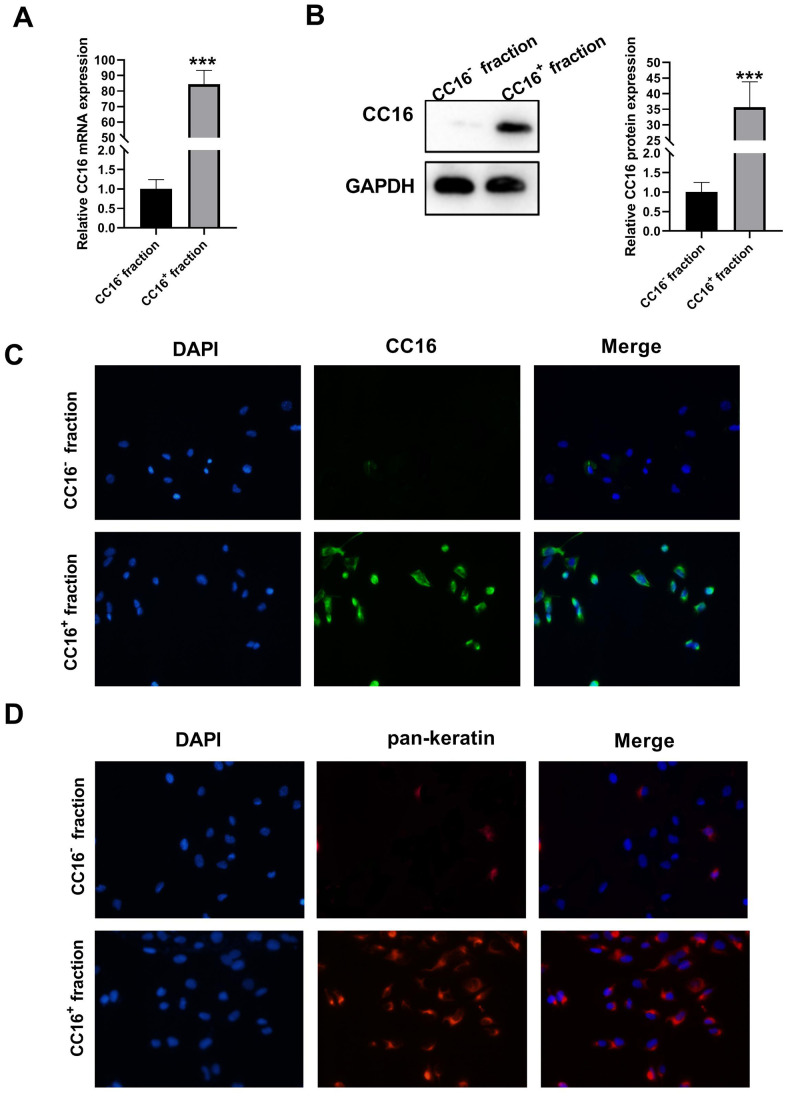
**Characterization of sorted cells.** (**A**, **B**) CC16 mRNA and protein expression in the sorted CC16^+^ and CC16^-^ cell fraction are detected by RT-PCR and western blot assay. (**C**, **D**) Immunofluorescence (IF) staining shows that 98% of the cells in CC16^+^ sorted fraction express CC16, and all the cells in CC16^+^ sorted fraction show positive expression of pan-keratin, the epithelial marker (× 100). ***P < 0.001 vs. CC16^-^ fraction.

Single cell suspensions were prepared by human distant normal lung tissues where Clara cells are located, and CC16^+^ and CC16^-^ cells were sorted directly by Fluorescence-activated cell sorting (FACS). The sorted CC16^+^ and CC16^-^ cells were cultured overnight, and unattached dead cells were removed from the medium. The CC16 mRNA and protein expression were subsequently detected. As shown in Figure 1A, 1B, CC16 mRNA and protein expression were sharply higher in CC16^+^ cell fraction than in CC16^-^ cells. We also performed immunocytochemistry staining of CC16 in sorted cells. As evidence by Figure 1C, 98% of the cells in CC16^+^ sorted fraction were positive for CC16 immunofluorescence (IF). In addition, to further confirm that the sorted CC16^+^ cells were distal airway Clara cells, the IF was performed for detection of pan-keratin expression, the epithelial marker. The results showed that all cells in the CC16^+^ sorted fraction expressed pan-keratin (Figure 1D). These data suggested that CC16-containing Clara cells are successfully isolated.

### DEP exposure induces C/EBPA hypermethylation and decreases CC16 production and secretion from CC16^+^ cells

**Figure 2:**
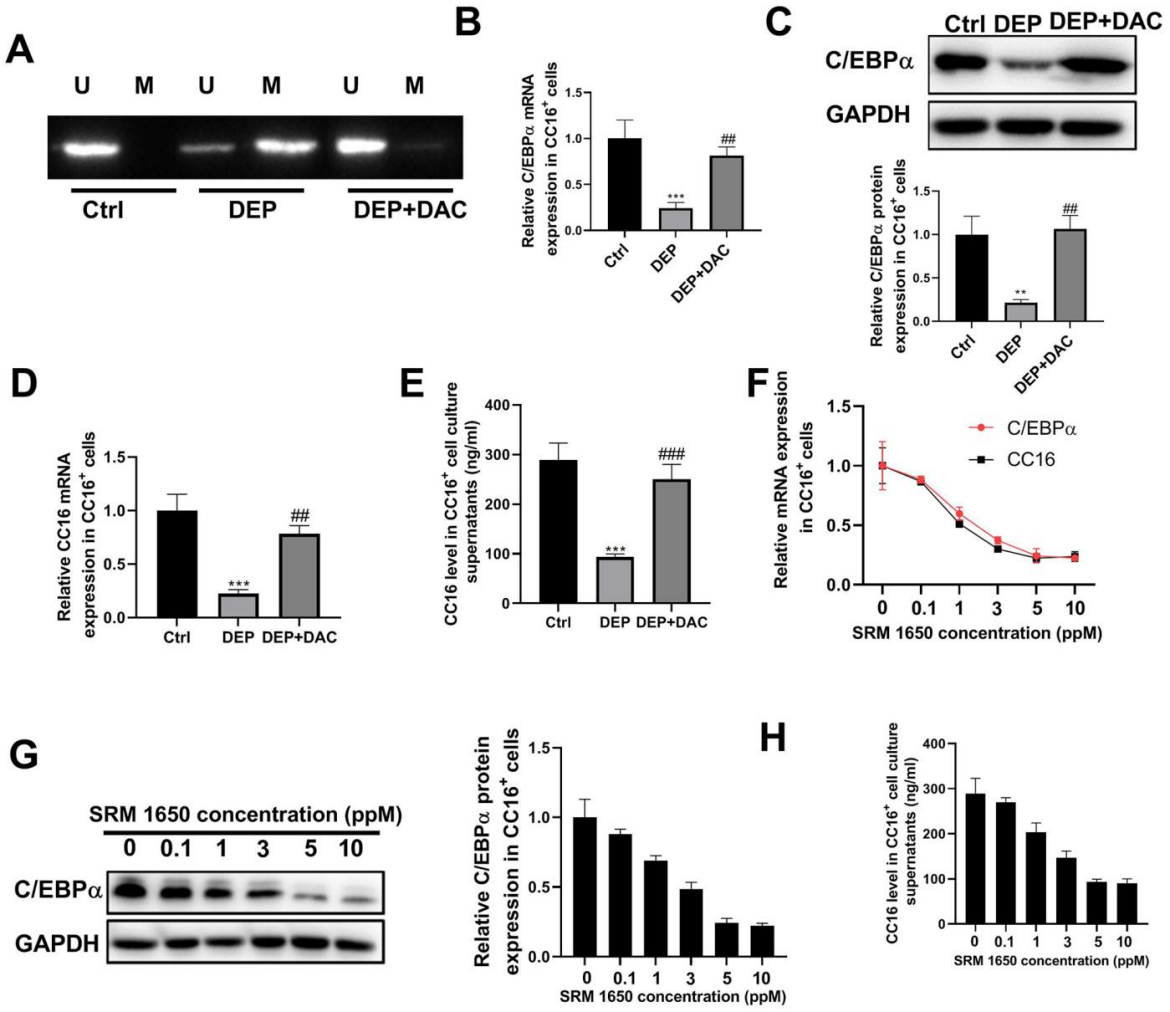
**DEP exposure induces C/EBPα hypermethylation and decreases CC16 production and secretion in CC16^+^ Cells.** CC16^+^ cells are exposed to 5 ppm SRM 1650b (the commercial DEP matter) in the presence of DAC or not for 48 h, and then cells and culture supernatants are collected. (**A**) MSP analysis is performed to detect the methylation of C/EBPα promoter. Lane U, amplified product with primers recognizing unmethylated C/EBPα sequence. Lane M, amplified product with primers recognizing methylated C/EBPα sequence. (**B**, **C**) The mRNA and protein expression of C/EBPα are determined by RT-PCR and western blot assay. (**D**, **E**) CC16 mRNA expression and level in culture supernatants are detected by RT-PCR and ELISA assay. (**F**–**H**) After treatment of CC16^+^ cells with different concentrations of SRM 1650b (0.1, 1, 3, 5 and 10 ppM), C/EBPα and CC16 mRNA expression, C/EBPα protein expression, and CC16 level in culture supernatants are determined by RT-PCR, western blot and ELISA assay. **P < 0.01 and ***P < 0.001 vs. Ctrl group; *^##^P < 0.01* and *^###^P < 0.001* vs. DEP group.

Our previous study showed that in C22 cells, DEP decreases CC16 expression by methylation of the C/EBPα promoter [10]. But it is unknown whether DEP regulates CC16 secretion via C/EBPα hypermethylation. The level of CC16 protein released into the culture media represents CC16 secretion capacity. The CC16^+^ cells were exposed to 5 ppm SRM 1650b (the commercial DEP matter) in the presence of demethylating agent 5-aza-2’-deoxycytidine (DAC) or not for 48 h. Then cells and culture supernatants were collected for methylation-specific polymerase chain reaction (MSP), RT-PCR, western blot and ELISA detection. The results of MSP determination showed that DEP treatment induced C/EBPα promoter methylation, while DAC treatment restored C/EBPα expression by demethylation of the promoter (Figure 2A). The results of RT-PCR and western blot showed that DEP treatment decreased C/EBPα mRNA and protein expression, which was significantly alleviated by DAC treatment (Figure 2B, 2C). Changes in CC16 level were consistent with changes in C/EBPα expression: DEP treatment significantly inhibited CC16 mRNA expression and level in culture supernatants, which were abolished by DAC treatment (Figure 2D, 2E). In addition, we also detected the effects of treatment with other concentrations of SRM 1650b (0.1, 1, 3 and 10 ppm) on C/EBPα mRNA and protein expression, and CC16 mRNA expression and level in culture supernatants. This can be seen in Figure 2F–2H that a dose-dependent inhibition of C/EBPα and CC16 by DEP was observed in CC16^+^ cells. Collectively, DEP exposure induces C/EBPα hypermethylation and decreases CC16 production and secretion in CC16^+^ cells.

### C/EBPA regulates CC16 secretion via Munc18b in CC16^+^ cells

**Figure 3:**
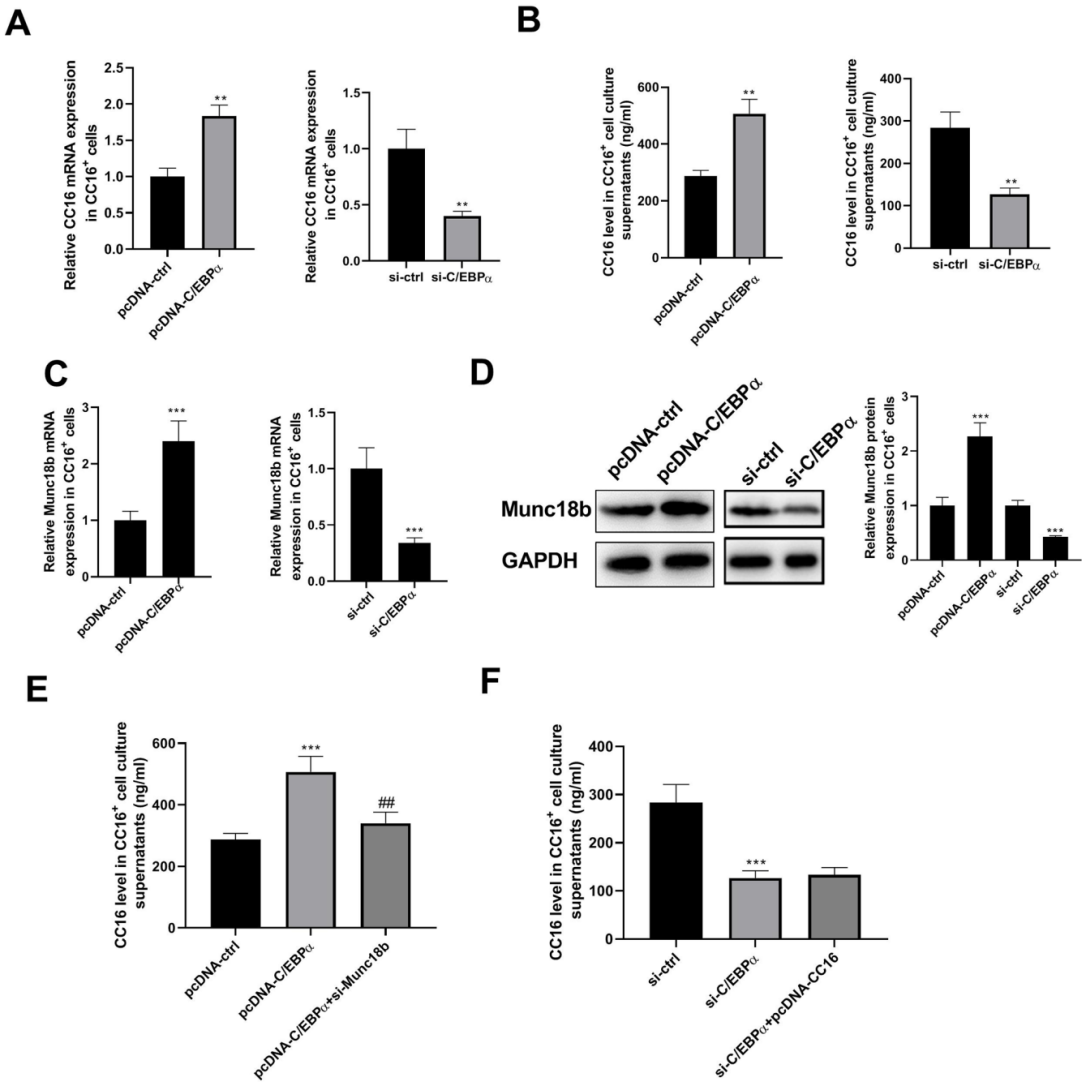
**C/EBPα regulates CC16 secretion via Munc18b in CC16^+^ cells.** After transfection of CC16^+^ cells with pcDNA-ctrl, pcDNA-C/EBPα, pcDNA-C/EBPα+si-Munc18b, si-ctrl, si-C/EBPα, or si-C/EBPα+ pcDNA-CC16, cells are cultured for 48 h, and then cells and culture supernatants are collected. (**A**, **B**) The effects of C/EBPα overexpression or knockdown on CC16 mRNA and protein released into the culture media are detected by RT-PCR and ELISA. (**C**, **D**) The effects of C/EBPα overexpression or knockdown on Munc18b mRNA and protein expression are detected by RT-PCR and western blot assay. (**E**, **F**) The increase of CC16 secretion induced by C/EBPα overexpression is abolished by Munc18b knockdown, while the decrease of CC16 secretion induced by C/EBPα knockdown is not affected by CC16 overexpression. **P < 0.01 and ***P < 0.001 vs. pcDNA-ctrl or si-ctrl group; *^##^P < 0.01* vs. pcDNA-C/EBPα group.

Our previous study demonstrated that in C22 cells, C/EBPα regulates CC16 expression [10]. Herein, we investigated whether and how C/EBPα regulates CC16 secretion. As shown in Figure 3A, 3B, C/EBPα overexpression caused the increases in CC16 mRNA and protein released into the culture medium, whereas C/EBPα knockdown resulted in the decreases of CC16 mRNA and secretion.

Munc18b is a limiting component of the exocytic machinery of secretory epithelial and immune cells. By searching the Jaspar database, we found that the 2-kb region upstream of Munc18b gene contains potential binding motifs for transcription factor C/EBPα in different species (human, rat and mouse) (Table 1). We thus examined whether C/EBPα regulates the transcription of Munc18b. As shown in Figure 3C, 3D, overexpression of C/EBPα significantly increased the expression of Munc18b mRNA and protein. As expected, knockdown of C/EBPα significantly reduced the expression of Munc18b mRNA and protein. In addition, the increase of CC16 secretion induced by C/EBPα overexpression was abolished by Munc18b knockdown, while the decrease of CC16 secretion induced by C/EBPα knockdown was not affected by CC16 overexpression (Figure 3E, 3F). These data suggested that C/EBPα promotes CC16 secretion through upregulation of Munc18b in CC16^+^ cells.

**Table 1:** The predicted binding sites between C/EBPα and the promoter region of Munc18b.

**Species**	**Matrix ID**	**Name**	**Score**	**Start**	**End**	**Predicted sequence**
Human	MA0102.3	CEBPA	5.139	-1715	-1705	gttacaaaagg
Human	MA0102.3	CEBPA	2.555	-1415	-1405	tttacaaaaca
Rat	MA0019.1	Cebpa	10.074	-1021	-1010	ggatgcaattgt
Mice	MA0102.1	Cebpa	8.717	-274	-263	ttgcccaagctt
Mice	MA0102.1	Cebpa	6.675	-361	-350	ttacagaatgtt
Mice	MA0102.1	Cebpa	6.335	-792	-781	atgtacaacagg

### C/EBPA can directly bind to the promoter region of Munc18b

**Figure 4:**
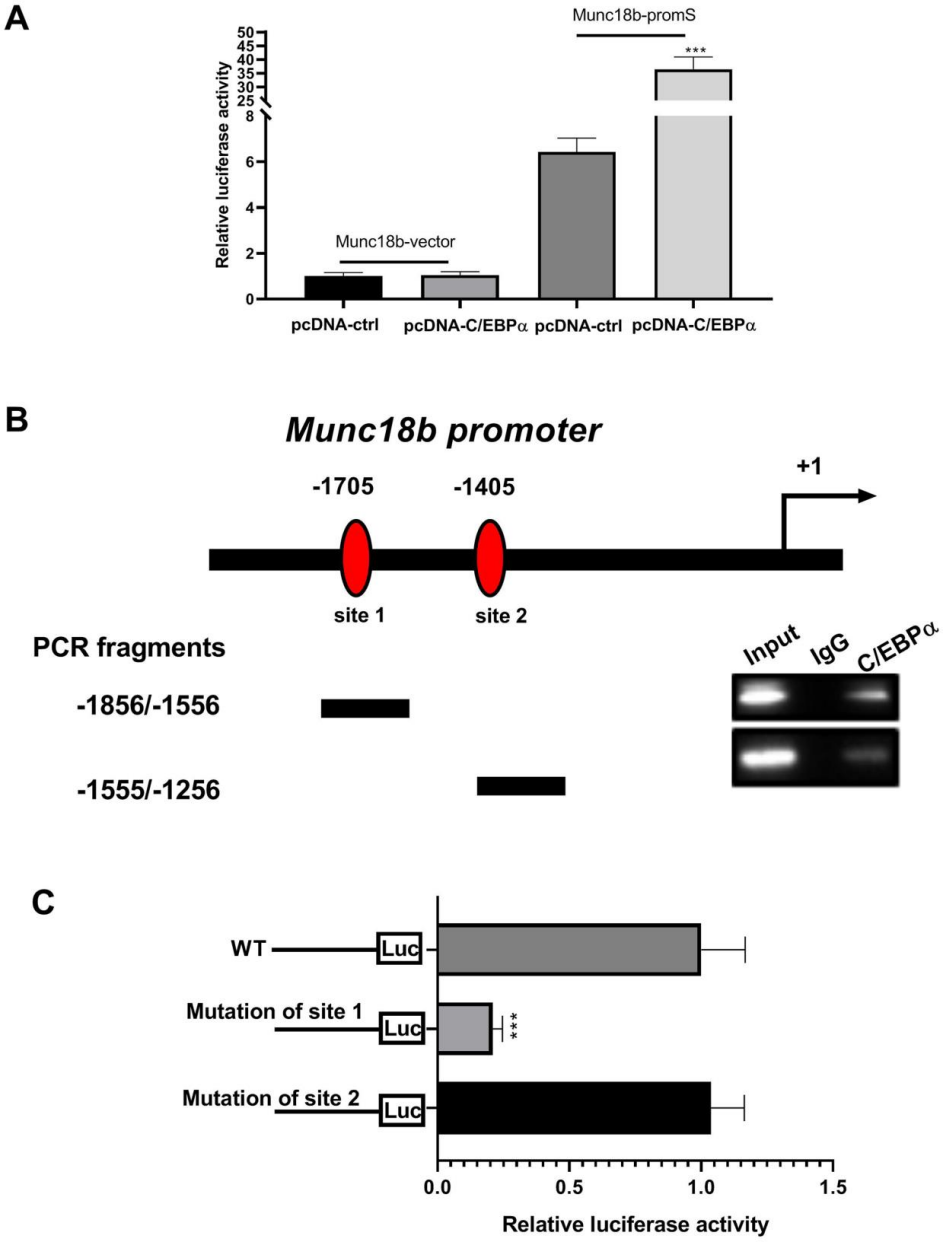
**C/EBPα can directly bind to the promoter region of Munc18b.** (**A**) CC16^+^ cells are transfected with both Munc18b promoter (-2000/200; Munc18b-promS) or empty pGL3-basic vector (Munc18b-vector) and C/EBPα plasmid or its corresponding empty vector control for 48 h, and then the cells are collected for luciferase activity detection. (**B**) ChIP assay is conducted in CC16^+^ cells with the anti-C/EBPα antibody. Interaction sites are identified by PCR for two possible C/EBPα binding sites in the Munc18b promoter. (**C**) Munc18b promoter with wild-type (WT) or mutation of the binding motif 1 or 2, and C/EBPα plasmid are co-transfected into CC16^+^ cells used in the luciferase assay. ***P < 0.001 vs. pcDNA-ctrl or WT group.

Given the above findings that the upstream of human Munc18b gene has two putative C/EBPα binding sites (-1705 and -1405 bp), and C/EBPα promotes Munc18b expression at the transcriptional level, we performed dual-luciferase reporter assay and chromatin immunoprecipitation (ChIP) assay to further investigate whether C/EBPα activates Munc18b transcription by directly binding to its putative promoter region. The luciferase activity was detected in CC16^+^ cells transfected with both Munc18b promoter construct (-2000/200; Munc18b-promS) or empty pGL3-basic vector (Munc18b-vector) and C/EBPα plasmid or its corresponding empty vector for 48 h. The results showed that Munc18b promoter construct led to an obvious increase in the relative luciferase activity, which was further reinforced by C/EBPα (Figure 4A). To identify the exact position where C/EBPα could bind within this fragment, a ChIP test was performed. As shown in Figure 4B, C/EBPα could bind to two putative binding sites, site 1 and site 2 sequences. Furthermore, to further clarify the function of site 1 and site 2, Munc18b promoter with wild-type (WT) or mutation of the binding motif 1 or 2, and C/EBPα plasmid were co-transfected into CC16^+^ cells. The results of the dual-luciferase reporter assay showed that in comparison with WT group, mutation of the binding motif 1 significantly inhibited the relative luciferase activity, and mutation of the binding motif 2 had no effect on the relative luciferase activity, suggesting that the site 1 was a functional site controlling Munc18b transcription (Figure 4C). These results indicated that C/EBPα activates Munc18b transcription by direct binding to the sites -1715/-1705 in Munc18b promoter.

### DEP induces bronchial epithelial cell autophagy and inflammation

**Figure 5:**
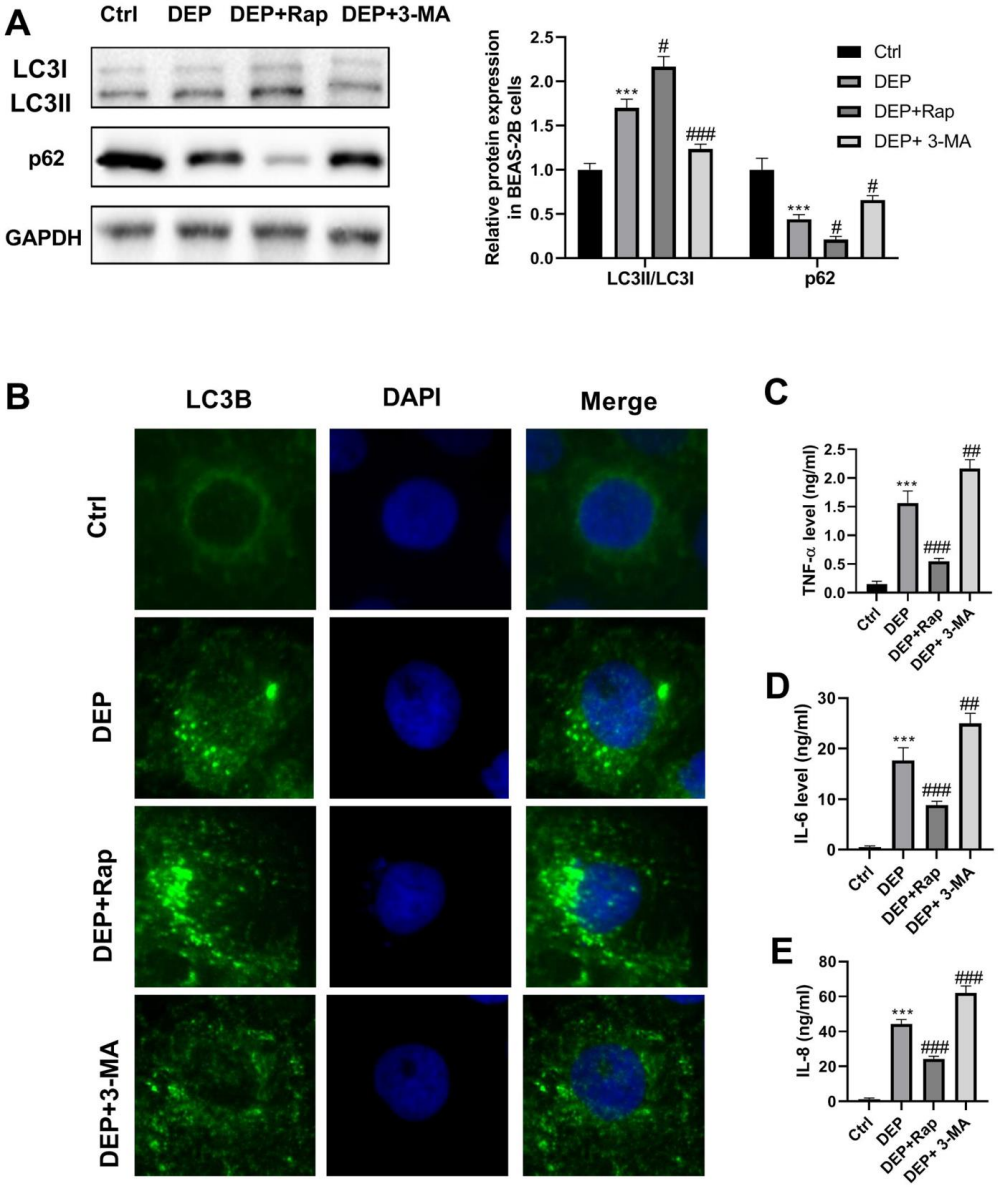
**DEP induces bronchial epithelial cell autophagy and inflammation.** BEAS-2B cells are pre-treated with 1.5 mM 3-MA or 10 nM Rap for 1 h, and then exposed to 5 ppm SRM 1650b. After 12 h, the cells and culture supernatants are collected. (**A**) Autophagy-related proteins LC3B and p62 expression in BEAS-2B cells are detected by western blot. (**B**) Autophagy induction is evaluated by LC3B immunostaining (× 400). Autophagosomes are identified as bright green dots. (**C**–**E**) Pro-inflammatory cytokines TNF-α, IL-6, and IL-8 level are detected by ELISA. ***P < 0.001 vs. ctrl; *^#^P < 0.05, ^##^P < 0.01*, and *^###^P < 0.001* vs. DEP group.

DEP has been widely reported to induce bronchial epithelial cell injury by regulation of autophagy, inflammation, oxidative stress, and apoptosis [27–30]. Here, we investigated the effects of DEP on autophagy and inflammation of BEAS-2B bronchial epithelial cells. LC3 and p62 are two proteins vital for autophagy. When autophagy is activated, soluble LC3-I is converted into lipid bound LC3-II, followed by being recruited to autophagosomal membranes, and thus the amount of LC3-II positively correlates with the number of autophagosomes. Furthermore, autophagosome formation enhances the autophagic flux, thereby accelerating p62 protein degradation. As evidenced by Figure 5A, DEP increased the ratio of LC3-II/LC3-I, and decreased p62 protein expression, which suggested that DEP treatment activated autophagy. DEP-activated autophagy was further reinforced by the autophagy promoter rapamycin (Rap), and relieved by the autophagy inhibitor 3-Methyladenine (3-MA). In addition, autophagosomes were identified as bright green dots by IF. The co-treatment group with Rap and DEP had the largest number of green spots, followed by DEP group, DEP+3-MA group, and ctrl group (Figure 5B). The effects of DEP exposure on pro-inflammatory cytokines were also detected. The results indicated that DEP exposure increased TNF-α, IL-6, and IL-8 level, which were relieved by Rap treatment, and aggravated by 3-MA treatment (Figure 5C–5E). These data suggested that enhancing autophagy can mitigate DEP-induced inflammation in BEAS-2B cells.

### CC16 secretion from CC16^+^ cells protects against DEP-induced bronchial epithelial cell inflammation

**Figure 6:**
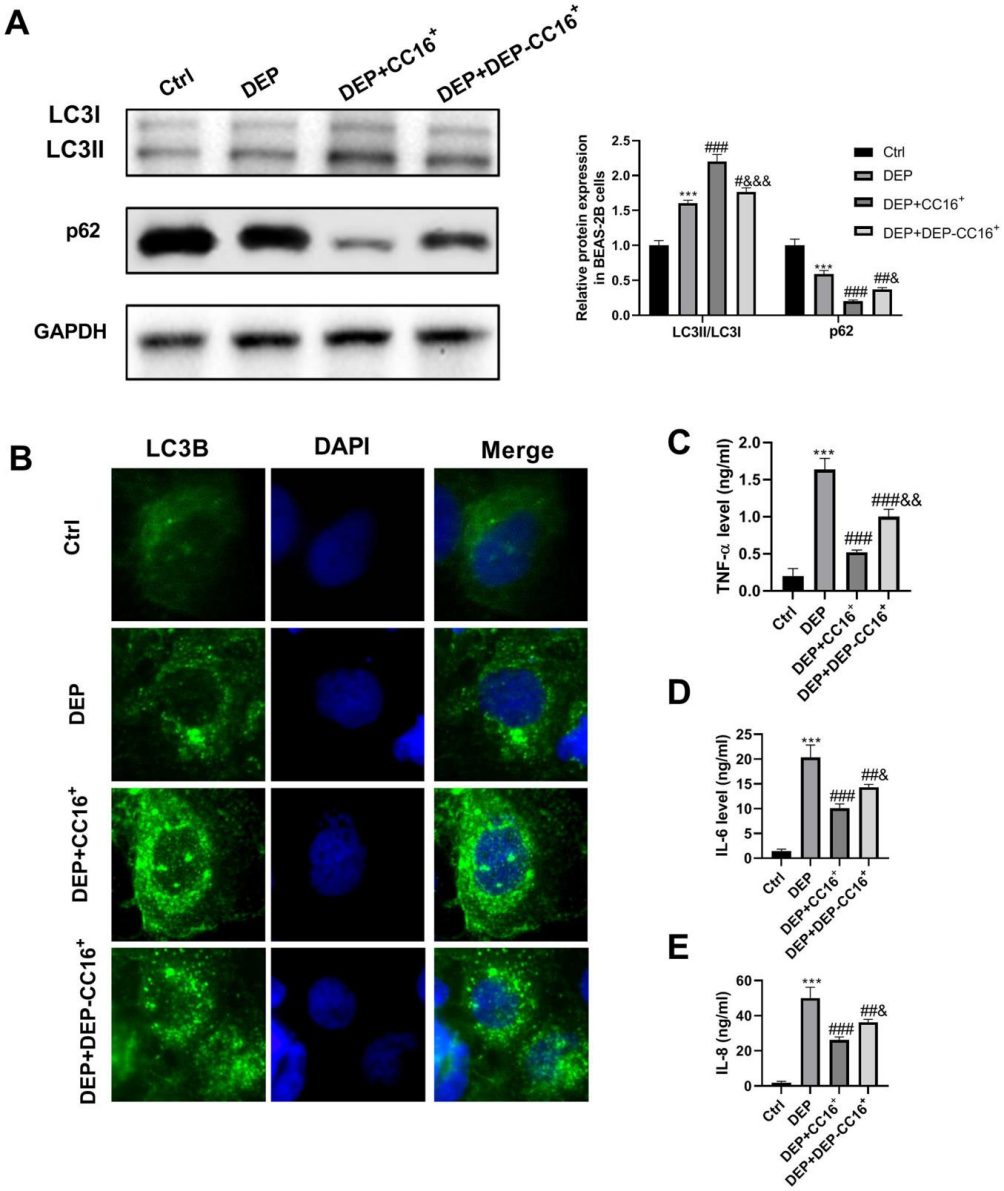
**CC16 secretion from CC16^+^ cells protects against DEP-induced bronchial epithelial cell inflammation via activation of autophagy.** CC16^+^ cell conditioned medium containing different concentrations of CC16 is prepared, and used to culture BEAS-2B cells. The CC16 level in the medium of Ctrl, DEP, DEP+ CC16^+^, DEP+ DEP-CC16^+^ group is 0, 0, 144.7 and 46.5 ng/ml, respectively. After 12 h of culture, the cells and culture supernatants are collected. (**A**) LC3B and p62 protein expression in BEAS-2B cells are detected by western blot. (**B**) Autophagy induction is evaluated by LC3B immunostaining (× 400). Autophagosomes are identified as bright green dots. (**C**–**E**) TNF-α, IL-6, and IL-8 level in culture supernatants are detected by ELISA. ***P < 0.001 vs. ctrl; *^#^P < 0.05, ^##^P < 0.01*, and *^###^P < 0.001* vs. DEP group; and *^&^P < 0.05, ^&&^P < 0.01*, and *^&&&^P < 0.001* vs. DEP+ CC16^+^ group.

**Figure 7:**
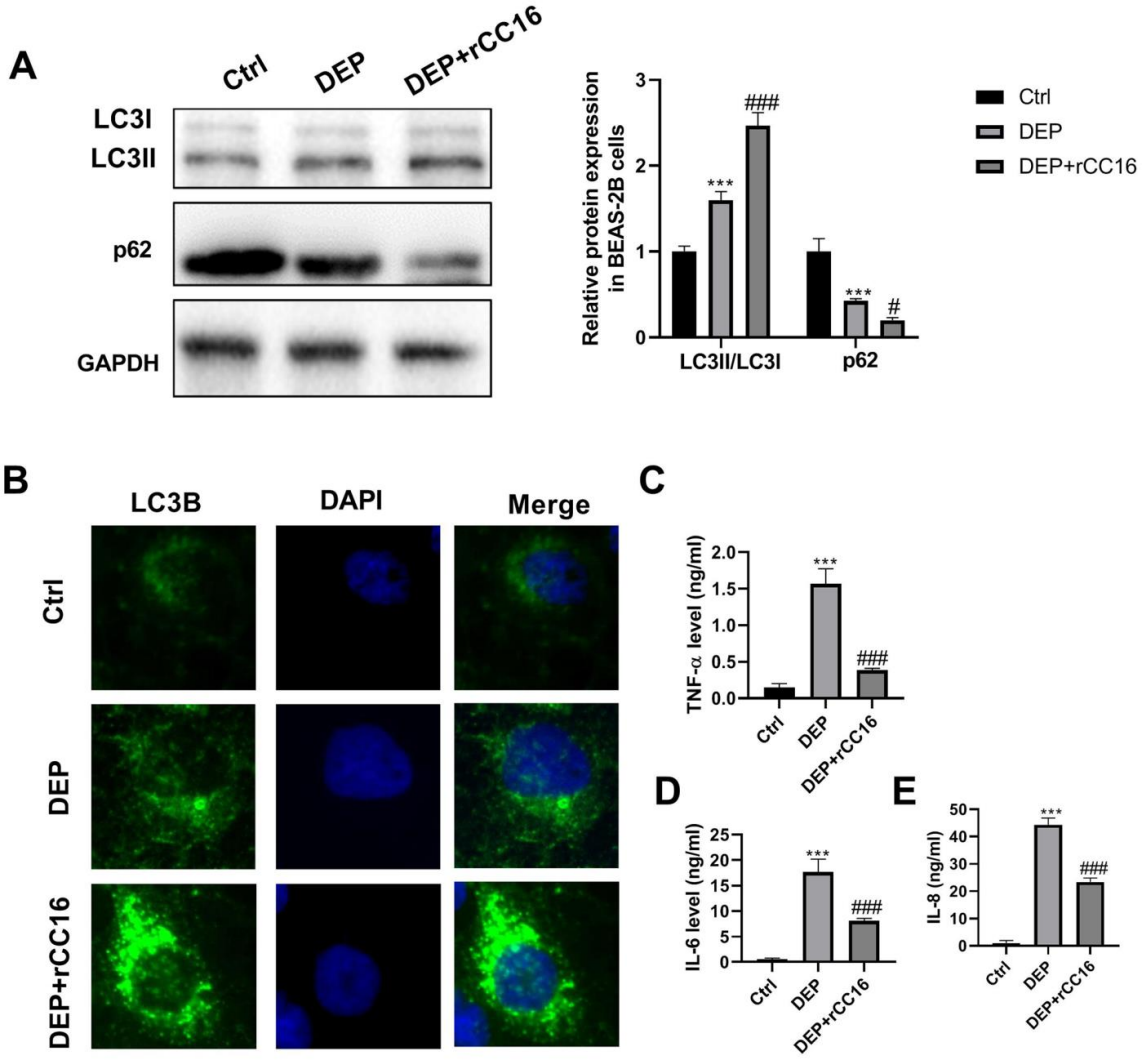
**rCC16 relieves DEP-induced bronchial epithelial cell inflammation via activation of autophagy.** BEAS-2B cells are incubated with 5 ppm SRM 1650b, or 300 ng/ml rCC16 + 5 ppm SRM 1650b for 12 h. Then the cells and culture supernatants are harvested. (**A**) LC3B and p62 protein expression in BEAS-2B cells are detected by western blot. (**B**) Autophagy induction is evaluated by LC3B immunostaining (× 400). Autophagosomes are identified as bright green dots. (**C**–**E**) TNF-α, IL-6, and IL-8 level in culture supernatants are detected by ELISA. ***P < 0.001 vs. ctrl; and #P < 0.05 and ###P < 0.001 vs. DEP group.

To investigate the effects of CC16 secretion from CC16^+^ cells on DEP-induced bronchial epithelial cell inflammation, CC16+ cells were cultured in DMEM/F12 medium with 5 ppm SRM 1650b or not to prepare CC16^+^ medium and DEP-CC16^+^ medium, respectively. After determining CC16 level, CC16^+^ cell conditioned medium were used to culture BEAS-2B cells. The CC16 level within the medium of ctrl, DEP, DEP+CC16^+^, DEP+DEP-CC16^+^ group was 0, 0, 144.7 and 46.5 ng/ml, respectively. Following exposure to DEP, autophagy and inflammation were detected. As shown in Figure 6, DEP+CC16^+^ group has the strongest autophagy activity, followed by DEP+DEP-CC16^+^ group, DEP group, and ctrl group. As expected, DEP induced the weakest inflammatory response in DEP+CC16^+^ group, followed by DEP+DEP-CC16^+^ group, and DEP group. CC16 level was positively correlated with DEP-induced autophagy activity, and negatively correlated with DEP-induced inflammatory response, suggesting that CC16 might mitigate DEP-induced inflammation via promoting autophagy in BEAS-2B cells. In order to further confirm that CC16^+^ cell conditioned medium functions in DEP-induced autophagy and inflammation mainly through CC16, BEAS-2B cells were incubated with 5 ppm SRM 1650b, or 300 ng/ml recombinant CC16 (rCC16)+5 ppm SRM 1650b for 12 h. The results showed that rCC16 increased DEP-induced autophagy and inhibited DEP-induced inflammation (Figure 7). Collectively, these data indicated that CC16 protects against DEP-induced bronchial epithelial cell inflammation via regulation of autophagy, and DEP-inhibited CC16 secretion from Clara cells exerts a decreased protection role in DEP-induced bronchial epithelial cell inflammation.

### TNF-α released by BEAS-2B cells induces CC16 production and secretion in CC16^+^ cells

**Figure 8:**
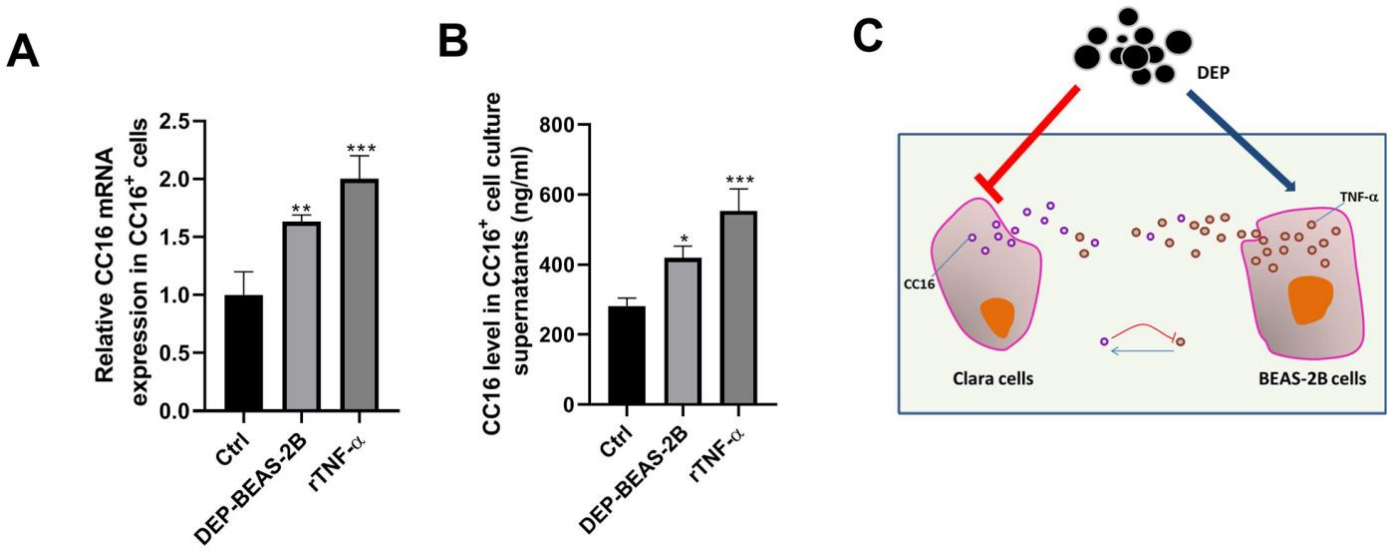
**TNF-α released by BEAS-2B cells induces CC16 production and secretion from CC16^+^ cells.** BEAS-2B cell conditioned medium (DEP-BEAS-2B) is prepared, and used to culture CC16^+^ cells. The TNF-α level in the culture medium of ctrl, DEP-BEAS-2B, and rTNF-α group is 0, 0.81 and 3 ng/ml, respectively. CC16^+^ cells are cultured in these media for 48 h. Then CC16 mRNA expression (**A**) and level in culture supernatants (**B**) are detected by RT-PCR and ELISA assay. (**C**) CC16-TNF-α negative feedback loop between Clara cells and normal airway epithelial cells protects against DEP exposure-induced injury. Red upside down T line drawing indicates inhibitive action. Blue line with arrowhead drawing indicates driving role. *P < 0.05, **P < 0.01 and ***P < 0.001 vs. Ctrl.

TNF-α plays a role in stimulating CC16 production [26]. Herein, we investigated whether BEAS-2B cell conditioned medium stimulates CC16 production and secretion mainly via TNF-α. As shown in Figure 8A, 8B, CC16 mRNA expression and level in culture supernatants were significantly increased when CC16^+^ cells were cultured in BEAS-2B cell conditioned medium containing 0.81 ng/ml TNF-α or the normal medium supplemented with 3 ng/ml recombinant TNF-α (rTNF-α). These data suggested that CC16 and TNF-α form a negative feedback loop: CC16 secreted by CC16^+^ cells inhibits TNF-α production in BEAS-2B cells, and TNF-α stimulates CC16 production and secretion, thereby protecting against DEP exposure-induced inflammation (Figure 8C).

## DISCUSSION

CC16 belongs to the secretoglobin family, and is a small anti-inflammatory and antioxidant protein secreted by non-ciliated epithelial Clara cells. Respiratory diseases are often accompanied by varying degrees of damage in the structural integrity and function of airway epithelium, while CC16 is a useful biomarker of airway epithelial damages in chronic respiratory diseases. Increasing evidence has confirmed that the number of CC16-positive epithelial cells is significantly decreased in allergic airway diseases such as asthma, allergic rhinitis and chronic rhinosinusitis, resulting in the lower level of CC16 in airway mucosa and bronchoalveolar lavage fluid (BALF) [31]. The baseline serum CC16 level is believed to be a predictive biomarker of lung function, which is decreased in COPD patients and is also correlated with smoking status [32, 33]. Another study documented that CC16-deficient mice have more severe airway inflammation than wild-type mice [34, 35]. In addition, the CC16 level in BALF is lower in the silicosis group than the control group and may have a specific reference value for the early diagnosis of silicosis [36, 37]. Our previous study also showed that CC16 level is decreased in serum and BALF in mice with DEP exposure-induced lung injury. How does DEP exposure reduce CC16 level? Our previous study only confirmed that DEP inhibits CC16 expression in C22 cells by methylation of the C/EBPα promoter [10]. In fact, CC16, a secretory protein, works after release into extracellular environment (also called secretion), such as the airway surface fluid and the culture media. However, the molecular mechanism by which DEP regulates CC16 secretion is not clear.

In this study, we firstly isolated CC16-containing Clara cells from human distal lung by a flow cytometric sorting method, followed by investigating the effects of DEP exposure on C/EBPα methylation, and CC16 production and secretion. Consistent with our previous study, DEP exposure induces C/EBPα hypermethylation, and decreases CC16 production and secretion from CC16^+^ Cells [10]. Notably, further exploration of the mechanism displayed that C/EBPα promotes CC16 secretion via activation of Munc18b transcription by direct binding to the sites -1715/-1705 in the Munc18b promoter.

Airway epithelial cells with an extremely low or no expression of CC16 represent most of the total epithelial cells. How does DEP affect these cells? The BEAS-2B cell line is a type of human airway epithelial cells. It has been reported that CC16 could not be detected in BEAS-2B cell lysates [38]. We also examined CC16 protein content in BEAS-2B cells and culture supernatants. Consist with the findings of Mark and colleagues [38], CC16 is not detected in BEAS-2B cells and culture supernatants (data not shown). As such, BEAS-2B cells were chosen and used for investigating the effects of DEP on airway epithelial cells with no CC16 expression.

Indeed, two decades ago, studies suggested that DEP induces pro-inflammatory responses in BEAS-2B cells. For example, in 1999, Takizawa et al. [23] reported that DEP induces pro-inflammatory cytokine transcription through NF-κB activation in BEAS-2B cells. Subsequently, Tal et al. [24] confirmed that low organic-containing DEP exposure stimulates pro-inflammatory cytokine IL-8 expression via activation of NF-κB-dependent of transcription. But high organic-containing DEP exposure induces IL-8 expression independently of NF-κB through a mechanism that requires Jun activity. In 1998, another study documented that DEP induces release of IL-6 and IL-8 by BEAS-2B cells [25]. NF-κB and Jun are both major mediators in autophagy. Autophagy is a cellular mechanism for the sequestration and degradation of intracellular pathogens and compromised organelles, and also clears other cellular components, such as inflammasomes and pro-inflammatory cytokines. In fact, DEP has been reported to activate autophagy in BEAS-2B cells [27]. These findings tempted us to speculate that autophagy is implicated in DEP-induced inflammation. We then co-treated BEAS-2B cells with DEP exposure and the autophagy promoter Rap or autophagy inhibitor 3-MA. As we expected, we found that DEP induces autophagy and the increases of pro-inflammatory cytokines TNF-α, IL-6, and IL-8 level, and co-treatment with Rap or 3-MA significantly reduces or increases the level of these three pro-inflammatory cytokines, respectively, suggesting that enhancement of autophagy can improve DEP-induced inflammation.

The above experimental results showed that DEP inhibits CC16 secretion from Clara cells along the C/EBPα-Munc18b axis, and induces the inflammatory response in BEAS-2B cells. We were curious about whether there are links between DEP-inhibited CC16 secretion in epithelial Clara cells and DEP-induced the inflammatory response in BEAS-2B airway epithelial cells without CC16 expression? Increasing evidence showed that CC16 protects airway epithelial cells from environmental stimuli, such as house dust mite and cigarette smoke exposure [20–22]. Herein, the role of CC16 in bronchial epithelial cells exposed to DEP was investigated. CC16^+^ cell conditioned media containing different concentrations of CC16 were prepared and used to culture BEAS-2B cells. We found a positive correlation between CC16 level and DEP-induced autophagy activity, and a negative correlation between CC16 level and DEP-induced inflammatory response, suggesting that CC16 might mitigate DEP-induced inflammation via promoting autophagy in BEAS-2B cells. We added rCC16 to BEAS-2B cells exposed to DEP, and further certified this finding. TNF-α has been reported as a boost to CC16 production [26]. Here, we also confirmed that DEP-induced TNF-α release from BEAS-2B cells stimulates CC16 production, suggesting that CC16 and TNF-α form a negative feedback loop.

## CONCLUSIONS

Our data showed that DEP inhibits CC16 secretion from CC16^+^ cells via methylation of C/EBPα and inhibition of Munc18b transcription; CC16 protects against DEP-induced BEAS-2B cell inflammation via activation of autophagy; the decreased CC16 level induced by DEP exerts a weaker protective role in DEP-induced inflammation; and DEP-induced TNF-α release from BEAS-2B cells stimulates CC16 production and secretion in Clara cells. Our findings revealed the CC16-TNF-α negative feedback regulatory mechanism for protection against DEP-induced inflammation. These findings prompt us to conduct more studies for understanding the delicate regulatory mechanisms, and to investigate the potential of manipulating CC16-TNF-α negative feedback against DEP-induced inflammation.

## MATERIALS AND METHODS

### Preparation of single-cell suspensions from human distal lung

All human lung tissues were obtained from patients who underwent pulmonary resection at the Qingdao Municipal Hospital. All patients provided informed consent, and the study was approved by the Qingdao Municipal Hospital Ethics Committee. Single cell suspensions of the human distal lung were prepared as previously described, with some modifications [39]. Briefly, human distant normal lung tissues were cut into small pieces, and incubated with DNase I (Sigma-Aldrich, St Louis, MO, USA; final concentration, 0.1 mg/ml), and collagenase (Roche Applied Science, Penzberg, Germany; final concentration, 3 mg/ml) for 1 h at 37° C with continuous agitation. Following incubation with 0.25% trypsin (Sigma-Aldrich) for 10 min, the suspensions were further disaggregated by trituration through needles (Sherwood Medical Co, St Louis, MO, USA), and filtered with a 100-μm mesh filter (BD Biosciences, San Jose, CA, USA). After washing with PBS, cells were treated with red blood cell lysis buffer (eBioscience, San Diego, CA, USA), filtered through a 40-μm mesh filter (BD Biosciences), resuspended in DMEM/10% fetal bovine serum (FBS; Invitrogen, Carlsbad, CA, USA) with 1% amino acid solution (Invitrogen), 2.5 mg/ml amphotericin B (Sigma-Aldrich), 100 U/ml penicillin, and 100 μg/ml streptomycin, and placed to recover in a humidified incubator at 37° C with 5% CO_2_ for 24 h.

### Flow cytometry

CC16-expressing cells (Clara cells) were obtained by FACS as previously described [39]. Briefly, the recovered cells were trypsinized, and resuspended into PBS with 3% FBS. Following incubation with an anti-rabbit CC16 antibody (Millipore, Billerica, MA, USA), an anti-rabbit-FITC secondary antibody was added and further incubated on ice for 30 min. After washing in PBS with 3% FBS, the cells were resuspended in the fresh medium. Isotype-matched rabbit IgG staining was used as a negative control, and CC16 staining with permeabilization of dissociated cells was used as a positive control. The cell fractions with minimal (CC16^-^ fraction) and strong (CC16^+^ fraction) expression of CC16 were obtained by a FACS Vantage SE cell sorter (Becton Dickinson, San Jose, CA, USA), and then examined by RT-PCR, western blot and IF.

### Immunofluorescence

Cells were fixed in 4% paraformaldehyde for 10 min at room temperature (RT), and permeabilized with 0.1% Triton X-100 for 10 min at RT. After blocking with 5% bovine serum albumin (BSA; Sigma-Aldrich) for 30 min, immunolabelling was performed via incubation of the cells with primary antibody CC16 (1:50, Santa Cruz Biotech, Santa Cruz, CA, USA), pan-keratin (1:100, Dako, Carpinteria, CA, USA), or LC3B (1:100, Cell Signaling Technology, Beverly, MA, USA) for 1 h in a humid chamber at RT, and subsequent incubation with secondary antibodies conjugated with Alexa Fluor 488 or 546 (Invitrogen) for 1 h. After washing three times in PBS, slides were mounted with an anti-fading reagent with 4’,6-diamidine-2-phenylindole (DAPI) (Invitrogen), air dried in the dark, and then observed under a fluorescence microscope (Olympus, Hamburg, Germany).

### RT-PCR

Total RNA was extracted from the cultured cells using Trizol reagent (Invitrogen) in accordance with the instructions of the manufacturer. The cDNA synthesis was performed by using Reverse Transcription Kit (Takara, Dalian, China), and then PCR was performed on SmartCycler® II System (Cepheid Inc., Sunnyvale, CA, USA). GAPDH was used as a reference. The relative quantification of transcripts was calculated by using the 2^-ΔΔCt^ method.

### Western blot analysis

Western blot assay was conducted as previously described [40]. The primary antibodies against pan-keratin (Abcam, Cambridge, MA, USA), CC16 (Millipore), C/EBPα (Santa Cruz Biotechnology, Santa Cruz, CA, USA), Munc18b (Abcam), LC3B (Abcam), p62 (Abcam), and GAPDH (Sigma-Aldrich), and horseradish peroxidase-conjugated secondary antibody (Abcam) were employed in this assay.

### ELISA

The cells were cultured under the indicated conditions, and culture supernatants were collected used for IL-6, IL-8, TNF-α and CC16 level determination. The levels of the cytokines IL-6, IL-8, and TNF-α were determined by the ELISA kits purchased from Bender Medsystems (Burlingame, CA, USA), and CC16 level was detected by the ELISA kit obtained from BioVendor (Brno, Czech Republic).

### Methylation-specific polymerase chain reaction

Genomic DNA was isolated from cultured cells using standard methods. Then DNA was modified with bisulfite by using CpGenome DNA modification kit (Intergen, Burlington, MA, USA), and then bisulfite-modified DNA (50 ng) was amplified in duplicate by MSP as described previously [41, 42]. The primer sequences for amplification were as follows: the methylated C/EBPα, sense primer: 5’-GTCGGGTATAAAAGTTGGGTCGGC-3’, and antisense primer: 5’-ATTCTCCCGACATAACGAACCTCG-3’; and unmethylated C/EBPα: sense primer: 5’-TTGTTGGGTATAAAAGTTGGGTTGGT-3’, and antisense primer: 5’-AAAATTCTCCCAACATAACAAACCTCA-3’. PCR products were subsequently separated by gel electrophoresis and visualized by ethidium bromide (EB; Sigma-Aldrich) staining.

### CC16^+^ cell culture and treatment

Isolated CC16^+^ cells were cultured in the serum-free DMEM/F12 medium supplemented with 20 ng/ml EGF (Invitrogen), bFGF (Invitrogen) and gentamicin (Sigma-Aldrich), 1 μl/ml bovine hypothalamus extract (PromoCell, Corning, NY, USA), 5 μg/ml transferrin (Roche Applied Science, Indianapolis, IN, USA) and insulin (RIA, Linco Research, St Charles, MO, USA), 1 mM retinol (Sigma-Aldrich) and hydrocortisone (Sigma-Aldrich), 1% penicillin-streptomycin (Invitrogen), and 1 ng/ml amphotericin B.

To assess whether DEP regulates CC16 production and secretion in CC16^+^ cells via induction of C/EBPα hypermethylation, CC16^+^ cells were treated with 5 ppm SRM 1650b (the National Institute of Standards and Technology, Gaithersburg, MD, USA) in the presence of DAC (Sigma-Aldrich) or not for 48 h. Then MSP, RT-PCR, western blot and ELISA were performed to detect the methylation of C/EBPα, the mRNA and protein expression of C/EBPα, CC16 mRNA expression, and CC16 level in culture supernatants, respectively. Additionally, we also determined the effects of treatment with other concentrations of SRM 1650b (0.1, 1, 3 and 10 ppm) on C/EBPα mRNA and protein expression, CC16 mRNA expression and level in culture supernatants.

To further investigate whether C/EBPα regulates the secretion of CC16 from CC16^+^ cells via Munc18b, CC16^+^ cells were transfected with the specific small-interfering RNAs targeting C/EBPα (si-C/EBPα), si-Munc18b, pcDNA-C/EBPα, pcDNA-CC16 or negative control alone or in combination using Lipofectamine 2000 (Invitrogen). The sequence fragment of C/EBPα or CC16 was amplified, sequenced, inserted into pcDNA3.1 vector (Invitrogen), and finally named as pcDNA-C/EBPα or pcDNA-CC16. si-C/EBPα, si-Munc18b and scrambled siRNA were synthesized by Sangon Biotechnology, Co., Ltd. (Shanghai, China). After transfection, the cells were collected used for determination of Munc18b mRNA and protein expression, and CC16 mRNA expression and secretion via RT-PCR, western blot and ELISA.

To investigate the effects of BEAS-2B cells treated with DEP on CC16 secretion from CC16^+^ cells, a BEAS-2B cell conditioned medium was prepared used for CC16^+^ cell culture. In addition, to determinate whether the BEAS-2B cell conditioned medium functions via TNF-α, CC16^+^ cells were treated with human rTNF-α (PeproTech, Rocky Hill, NJ, USA) for 48 h. Then CC16 mRNA expression in the cells and protein level in culture supernatants were determined.

### Dual-luciferase reporter assay

To investigate whether C/EBPα regulates Munc18b expression via binding to the promoter of Munc18b, the upstream regions (-2000/200) of Munc18b gene with WT or mutation of the binding motifs 1 or 2 with C/EBPα were amplified and subcloned into the pGL3-Basic vector by GenePharma (Shanghai, China) to generate Munc18b promoter constructs. The cells were co-transfected with Munc18b promoter construct or empty pGL3-basic vector and C/EBPα plasmid or its corresponding empty vector. The cells were then lysed and analyzed with Dual luciferase reporter assay system (Promega, WI, USA).

### Preparation of CC16^+^ or BEAS-2B cell conditioned medium

CC16^+^ cells were cultured in DMEM/F12 medium containing 5 ppm SRM 1650b or not for 48 h. Then the medium was harvested, cleared by centrifugation, diluted 1:1 with F12 medium, stored at 4° C and named as CC16^+^ medium and DEP-CC16^+^ medium. As well, the BEAS-2B cell conditioned medium was prepared.

### BEAS-2B cell culture and treatment

A human bronchial epithelial cell line BEAS-2B (American Type Culture Collection, Manassas, VA, USA) was cultured in hormonally defined Ham’s F12 medium (HD-F12) supplemented with 1% penicillin-streptomycin, 5 μg/ml insulin and transferrin, 25 ng/ml EGF, 15 μg/ml endothelial cell growth supplement (Collaborative Research, Inc., Bedford, MA, USA), 200 pM triiodothyronine (Gibco, Carlsbad, CA, USA), and 1 mM hydrocortisone.

To investigate whether DEP induces bronchial epithelial cell inflammation via autophagy, BEAS-2B cells were pre-treated with 1.5 mM 3-MA (Sigma-Aldrich) or 10 nM Rap (Sigma-Aldrich) for 1 h, and then exposed to 5 ppm SRM 1650b for 12 h. Western blot, IF, and ELISA were then applied to detect the autophagy-related proteins LC3B and p62 expression, and pro-inflammatory cytokines TNF-a, IL-6, and IL-8 level in culture supernatants.

To investigate whether CC16 secretion from CC16^+^ cells protects against DEP-induced bronchial epithelial cell inflammation, BEAS-2B cells were divided into four groups and cultured with different medium for 12 h, including ctrl group (equiproportional mixing of fresh DMEM/F12 and HD-F12), DEP group (the same medium with ctrl group but containing 5 ppm SRM 1650b), DEP+CC16^+^ group (CC16^+^ medium containing 5 ppm SRM 1650b), and DEP+DEP-CC16^+^ group (DEP-CC16^+^ medium containing 5 ppm SRM 1650b). Next, the expression of p62 and LC3B protein, and the levels of TNF-a, IL-6, and IL-8 level were determined by western blot, IF, and ELISA, respectively.

For further confirmation the protection role of CC16 against DEP-induced bronchial epithelial cell inflammation, BEAS-2B cells were exposed to 5 ppm SRM 1650b in the presence of rCC16 (prepared by our laboratory) or not for 12 h. Then autophagy and inflammation were determined.

### Chromatin immunoprecipitation

ChIP assay was conducted by using a ChIP assay Kit (Upstate Biotechnology, Billerica, MA, USA) as per the manufacturer’s instructions. Briefly, the cells were harvested, fixed with 1% formaldehyde for 10 min at RT, and resuspended in lysis buffer containing a protease inhibitor cocktail. Following sonication, the soluble chromatin was diluted with ChIP-dilution buffer and immunoprecipitated with C/EBPα antibody or IgG as a negative control overnight at 4° C. Then the chromatin-antibody complexes were eluted with the elution buffer. After reversion of the cross-link, the DNA was purified by Qiaquick PCR product Purification Kit (QIAGEN Inc, Chatsworth, CA, USA). End-point PCR was conducted to amplify the site 1 and 2 (two potential binding motifs for C/EBPα) by using primers spanning the C/EBPα binding elements in the promoter region of the Munc18b gene.

### Statistical analysis

All statistical analyses were performed with GraphPad Prism 8. The data were presented as the mean ± standard deviation (SD) from at least three independent experiments. The comparisons between groups were done using the Student’s *t* test or ANOVA, and *P* < 0.05 was considered to be statistically significant.
